# M-Mode Ultrasound Examination of Soleus Muscle in Healthy Subjects: Intra- and Inter-Rater Reliability Study

**DOI:** 10.3390/healthcare8040555

**Published:** 2020-12-11

**Authors:** Carlos Romero-Morales, Cesar Calvo-Lobo, Emmanuel Navarro-Flores, Victoria Mazoteras-Pardo, Paula García-Bermejo, Daniel López-López, Eva María Martínez-Jiménez, Blanca De-la-Cruz-Torres

**Affiliations:** 1 Faculty of Sport Sciences, Universidad Europea de Madrid, Villaviciosa de Odón, 28670 Madrid, Spain; carlos.romero@universidadeuropea.es (C.R.-M.); paulagarber91@hotmail.com (P.G.-B.); 2 Facultad de Enfermería, Fisioterapia y Podología, Universidad Complutense de Madrid, 28040 Madrid, Spain; cescalvo@ucm.es; 3 Frailty Research Organized Group (FROG), Department of Nursing, Faculty of Nursing and Podiatry, University of Valencia, 46001 Valencia, Spain; 4 Grupo de Investigación ENDOCU, Departamento de Enfermería, Facultad de Fisioterapia y Enfermería, Universidad de Casilla la Mancha, 45004 Toledo, Spain; victoria.mazoteras@uclm.es (V.M.-P.); eva.martinez@uclm.es (E.M.M.-J.); 5 Research, Health and Podiatry Group, Department of Health Sciences, Faculty of Nursing and Podiatry, Universidade da Coruña, 15403 Ferrol, Spain; daniellopez@udc.es; 6 Deparment of Physiotherapy, University of Seville, Avicena Street, 41009 Seville, Spain; bcruz@us.es

**Keywords:** ultrasound, M-mode, soleus, reliability

## Abstract

Objective: M-mode ultrasound imaging (US) reflects the motion of connective tissue within muscles. The objectives of this study were to evaluate inter-rater and intra-rater reliability of soleus muscle measurements between examiners with different levels of US experience in asymptomatic subjects and to investigate the level of soleus muscle isometric activity in two positions (knee extended and knee flexed at 30°). Methods: Thirty volunteers without a history of ankle pain were evaluated with US examinations of the soleus muscle. Each muscle was scanned independently by two evaluators. Muscle at rest thickness, maximal isometric contraction thickness, time and velocity measures were detailed and blinded to the other examiner. Results: Intra- and inter-rater reliability at rest, in maximal isometric contraction thickness, contraction time and contraction velocity measures for both positions (extended and flexed knee) were reported from good to excellent for all outcome measurements. The position with the knee extended reported a statistically significant increase in thickness after motion showing 1.33 ± 0.27 mm for measurements at rest thickness with knee extended versus 1.50 ± 0.29 mm for measurements at end thickness with the knee in flexed position (*p* = 0.001), as well as 1.31 ± 0.23 mm for rest thickness with the knee in flexed position measurements with respect to 1.34 ± 0.24 mm for maximal isometric contraction thickness with extended knee measurements (*p* = 0.058). Conclusions: This study found that intra- and inter-examiner reliability of M-mode ultrasound imaging of the soleus muscle was excellent in asymptomatic subjects and the soleus muscle activity was different between the position with the knee extended and the position with the knee flexed.

## 1. Introduction

The triceps surae is the key muscle for the plantar flexor movement of the ankle [1]. This interesting muscle group is very relevant for biomechanical studies due to all three triceps surae muscle components having a great functional importance for performance in most sports and in the performance of activities of daily life [2,3,4]. Additionally, the activation of the gastrocnemius and soleus remains still unclear. For example, the position of the knee may drastically influence the force production of these muscles due to the length–tension relationship of each one [5]. In this line, the literature is controversial; for example, Pereira et al. [6] and Segnorine et al. [7] found that, under measurements with the knee flexed at 90°, the gastrocnemius muscles were under-activated, increasing the role of the soleus muscle. In addition, Dixon [8] suggested that the soleus muscle could be injured while the knee is flexed, while the gastrocnemius might be injured with the knee placed in a more extended position. Nevertheless, Hali et al. [9] showed that gastrocnemius activation was inhibited during plantar flexion contractions in a flexed position compared to an extended knee joint position, although this knee joint position change did not affect the soleus excitability, and Hëbert-Losier et al. [10] indicated that the muscle changes in gastrocnemii and soleus activities due to the different knee positions might not be enough to significantly influence clinical outcome measures or muscle-specific benefits.

Ultrasound (US) imaging is a noninvasive, safe and valid method to measure muscle and tendon morphology, such as cross-sectional area tendon [11], textural feature tendon [12], muscle shape [13], muscle thickness [14], fascicle length [15] and pennation angle with graded activity [16]. Specially, M-mode US shows the motion of connective tissue within muscles. As a muscle contraction is accompanied by a motion of muscle tissue, M-mode US has been used to explore the muscle activity onset in musculoskeletal structures, such as lumbar multifidus [17], abdominal [18], diaphragm [19] and gluteus medius and minimus [20,21]. Vasseljen et al. [17,18] explored whether M-mode US could measure muscle response onset in the lumbar multifidus and abdominal muscle compared with intramuscular electromyography, showing good reliability. Thus, the authors found that both procedures may be compared but with a small systematic delay for onset evaluation by M-mode ultrasound that should be corrected. Nevertheless, Scarlata et al. [19] showed that diaphragmatic thickness measurements using M-mode US were reproducible. In the same line, Dieterich et al. [20,21] assessed the activity of gluteus minimus and gluteus medius during different tasks and suggested that M-mode US was able to detect onset muscle responses of both muscles. The relationship between the level of isometric activity and the change of muscle thickness was different between muscles [22,23]. To the best our knowledge, this was the first study to assess and analyze the isometric activity of the soleus muscle to date. The evaluation of muscle contractions during different positions or movements provides to the therapist a better understanding about the muscle activity and features in dynamic situations. The authors hypothesize that M-mode US may use visual real-time information as a biofeedback in control motor approaches, as a guiding tool in clinical decisions, as well as to improve the understanding of tissue adaptations to exercise or movement. Nevertheless, according to the use of this method in clinical therapy and research, the reliability of US imaging is a key evaluation due to US maybe being operator-dependent, and the measurement protocol may influence the results. The authors considered that a proper intra- and inter-rater reliability may be essential to know the confidence details for any tool. Inter-rater is the degree of agreement among raters, and intra-rater reliability is a score of the consistency in ratings given by the same person across different times. In fact, there are many studies that mostly investigated the reliability of imaging calculations, and even reliability, according to the experience of the examiner, but all of them were made in B-mode US. [24,25] To date, M-mode US needs further research in order to provide reliable examination protocols and procedures [19].

Thus, the objectives of this study were to investigate the level of soleus muscle isometric activity in two positions: at knee extended and knee flexed at 30° by M-mode US, measuring the change of muscle thickness and, secondly, to analyze the intra- and inter-examiner reliability of M-mode imaging of soleus muscle thickness in healthy subjects, measured by an experienced or novice examiner.

## 2. Methods

### 2.1. Design

The present study was a reliability study, according to the Standards for the Reporting of Diagnostic Accuracy Studies (STARD) guidelines and checklist [26].

### 2.2. Participants

Thirty asymptomatic volunteers (12 female, and 18 male) without ankle pain symptoms were recruited from a physiotherapy care center (Seville, Spain) (Table 1). To be eligible to participate, they presented an age range between 18 and 55 years old and with no history of lower limb pain during the previous year. Exclusion criteria included a history of asymmetry limb, prior history of lower extremity surgery, prior history of ankle strain, lumbar radiculopathy or health problems that may affect normal muscle performance. A medical doctor of the center with more than 20 years of experience determined if the patients met the inclusion criteria for the study.

### 2.3. Ethical Considerations 

The present study was developed in accordance with the fundamental guidelines for clinical research in humans of the Declaration of Helsinki, and it was evaluated and approved by the Ethics Committee of Virgen de la Macarena and Virgen del Rocío Hospitals (Register code: ACT062019, 19 June 2019). Before the procedure, all the participants read and signed the informed consent form.

### 2.4. Procedure Assessment

**Figure 1:**
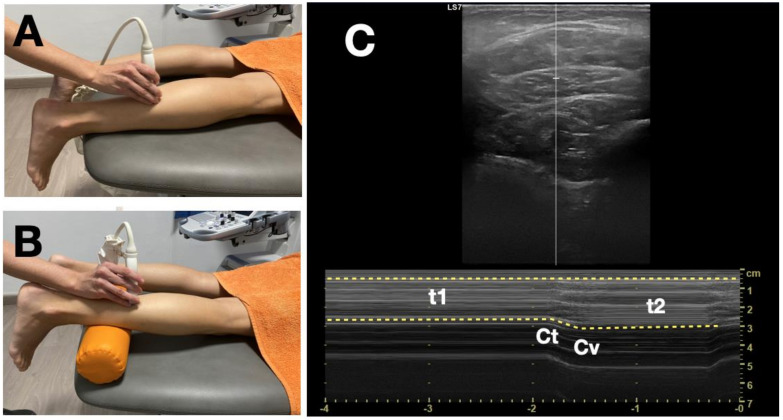
(**A**) Participants with their knees extended. (**B**) Participants with their knees flexed at 30°. (**C**) Ultrasound image of the soleus muscle displayed in M-mode during maximal isometric ankle plantar flexion measurements: t_1_, initial thickness; t_2_, end thickness; Ct, contraction time and Cv, contraction velocity.

An US machine (S7, GE Healthcare, Milwaukee, WI, USA) with a GE ML6-15 linear probe was used. According to a previous study [27], soleus US imaging was obtained in the distal third distance of the calf length. For this evaluation, the soleus muscle activity was measured in two positions: first, individuals were placed in the prone position, with their knees extended and ankle dorsiflexion at 0 degrees (Figure 1A), and second, participants were placed in the prone position, with a pillow underfoot, knees flexed at 30 degrees and ankle dorsiflexion at 0 degrees, measured with a classic goniometer (Baseline, Yarmouth, ME, USA) (Figure 1B). The soleus muscle was firstly identified in a B-model ultrasound and scanning angle adjusted to delineate thin, clear fasciae and intramuscular connective tissue. To record muscle motion during exercise performance, the ultrasound system was set to M-mode at the highest sweep speed, 2.44 s, providing a temporal resolution of 2.2 ms per pixel. Following three trials and a break of 10 s, maximal isometric right ankle plantar flexion, sustained for 3 s, was recorded in self-determined activation speed (Figure 1C). Visual feedback of the actual force exerted and target force level were provided.

For the ultrasound imaging assessment, the subjects were placed in the prone position to allow the establishment of standardized measurement protocols in relation to clinical variables, such as subject positioning, cursor location and transducer pressure, without the added variable of muscle contractions. In order to avoid the possible influences of pain and muscle inhibition, healthy participants were selected.

Two evaluators carried out the procedure assessment, including an experienced examiner with 10 years of experience in ultrasound imaging evaluation and a novel examiner with 1 year of experience. In order to provide the same experimental conditions, both evaluations were carried out in the same day. Both examiners captured images of the soleus muscles as described previously, with a 10-min period between evaluators. Additionally, the experienced examiner repeated the capture in a 10-min period. Participants were repositioned for each assessment, and a M-mode image of the soleus muscles was obtained. The final scores were collected by the mean of 3 repeated values for each measurement. Both evaluators developed the calculations of each image, and all the images were codified in order to blind the examiners by using numerical codes.

### 2.5. Clinical Measurements

Demographic data were obtained, including gender, age, weight, height and body mass index (BMI). US variables were measured using each machine’s measuring software. Rest thickness (t_1_), maximal isometric contraction (MIC) thickness (t_2_), contraction time (Ct) and contraction velocity (Cv) were obtained as ultrasound variables for 2 positions. The t_1_ (cm) was considered the distance between superficial and deep fascia of the soleus muscle at rest, the t_2_ (cm) was considered the distance between superficial and deep fascia of the soleus muscle during isometric exercise, Ct (seconds) was considered the time from t_1_ to t_2_ during the exercise and Cv (cm/seconds^2^) was considered the speed the muscle reaches t_2_.

### 2.6. Statistical Analysis

SPSS software (v.22, IBM, Armonk, NY, USA; IBM Corp) was employed for the statistical analysis. To check the normality assumption, a Shapiro-Wilk test was used. A 2-way, mixed-model, consistency-type, intra-class correlation coefficient (ICC) was calculated to assess the intra- and inter-rater reliability for each variable. Reliability was described as fair (ICC < 0.50), moderate (0.50 < ICC < 0.75), good (0.75 < ICC < 0.90) or excellent (ICC > 0.90). [27] Moreover, the ICC and standard deviation (SD) were employed to calculate the standard error measurement (SEM) as an evaluation of the precision of measurement, considered a good practice and widely employed in the clinical literature: SEM = SD × (√1−ICC). [28] Moreover, the smallest real difference (SRD) was calculated as 1.96 × SEM × √2. A comparative analysis between 0 and 30 knee flexion degrees was performed. Thus, the parametric data was analyzed employing a Student’s *t*-test for independent samples, and for the nonparametric data, the U Mann-Whitney test was used. An α error of 0.05 and a β error of 0.2 for both intra- and inter-examiner/rater reliability were employed through the study.

## 3. Results

Intra- and inter-rater reliability at rest, in maximal isometric contraction thickness, contraction time and contraction velocity variables for both positions, with knee extended and flexed, reported good to excellent reliability for all measurements (Table 2 and Table 3). Table 4 showed a measurement comparison between both positions at the knee straight and knee flexed at 30°. There were not significant baseline differences between both positions for each outcome measurement. Nevertheless, there were significant changes in the thickness, contraction time and contraction velocity after the muscle contraction in the maximal ankle dorsiflexion. These changes were due to the position with the knee extended reporting a statistically significant increase in thickness after the motion, showing 1.33 ± 0.27 mm for measurements at rest thickness with the knee extended versus 1.50 ± 0.29 mm for measurements at end thickness with the knee in the flexed position (*p* = 0.001), as well as 1.31 ± 0.23 mm for rest thickness with the knee in the flexed position measurements with respect to 1.34 ± 0.24 mm for maximal isometric contraction thickness with extended knee measurements (*p* = 0.058). The SRD oscillated between 0.156 for intra-rater test thickness and 1.568 for inter-rater contraction velocity at 0 knee flexion degrees. At 30 knee flexion degrees, the SMR oscillated between 0.133 intra-rater contraction time and 1.748 for intra-rater contraction velocity.

## 4. Discussion

The main findings of this study were that, firstly, the muscle activity onset in the soleus muscle measured by M-mode US was reliable. Secondly, the intra- and inter-rater reliability of M-mode US imaging of the soleus muscle was excellent in healthy subjects, independently of the examiner experience. The relevance of these findings into the clinical practice was evidenced due to that the M-mode US imaging assessment is not tester-dependent, according to how there was not differences between an experienced and novice assessor. Lastly, the soleus muscle activity was different between the position with the knee extended and position with the knee flexed. Muscle contraction of the soleus muscle showed also a thickness increase. Only a significant increase was observed in the thickness of the soleus muscle when participants presented the knee located in an extended position. Therefore, there were significant differences between both positions with respect to the maximal isometric contraction thickness, contraction time and contraction velocity. The authors hypothesized that, when subjects performed ankle dorsiflexion with the knee extended, soleus activity was higher, which may indicate motion from gastrocnemius during voluntary contractions. The data from this study were in line with other previous studies that recommend a select knee position for assessing, training and rehabilitating the soleus [9]. To the best of our knowledge, this is the first study to analyze the soleus muscle activity by M-mode US.

The onset of muscle activity is commonly recorded using electromyography (EMG). Usually, EMG allows to extract information that is often considered to be a global measure of motor unit activity [29,30]. Nevertheless, the surface EMG signal is susceptible to interference from the signal originated in mainly small, deep muscles [31,32]. Recent studies [18,19,33] have compared that the record of the onset of activation may be estimated from the muscle excitation registered by EMG and muscle movement recorded by ultrasound, which allows noninvasive measurements in the deep muscles. Dieterich et al. reported that M-mode ultrasound imaging can reflect the muscle activation closely related to EMG in both gluteus medius and gluteus minimus muscles with 100% SD800 and 96% SD800 onsets, respectively [34]. Tweedle et al. showed values with high variability between EMG and M-mode ultrasound imaging of the vastus lateralis and biceps brachii muscles: ICC = 0.65 and ICC = 0.40 [35]. Thus, authors indicated that the ultrasound detected onset before the EMG method; however, despite the high variability of the values, the results should be interpreted with caution. In this context, Vasseljen et al. [17] suggested that, if the researchers use an US M-mode imaging with high resolution, this may detect muscle activity comparably accurate to intramuscular EMG, reporting excellent ICC for the EMG (0.99) and, also, for the ultrasound imaging, with results similar to the findings of the present study (0.98). According to this, they all concluded that M-mode US may be used to measure noninvasively the onset of deep muscle activity. In our study, the authors used an US machine at a high time resolution (S7, GE Healthcare, Milwaukee, WI, USA). In this line, the results of this article showed good to excellent reliability of M-mode US imaging of the soleus muscle in both positions, and they corroborated that the knee position may influence the activity of the soleus muscle, such as in previous studies [6,7,8]. Further studies are needed to investigate the applicability of the US method in clinical practice.

They suggested that the data between both methods may vary secondary to electrical and mechanical activation onsets that appear to be influenced by modifying factors that could differ between muscles. Although all of them concluded that M-mode US may be used to measure noninvasively the onset of deep muscle activity, further studies are needed to explore the applicability of the ultrasound method in clinical settings.

This study may offer a novel approach to analyze and quantify the soleus muscle activity that could normally present alterations in active people, and these findings may guide clinicians in the use of M-mode US for rehabilitation of the soleus by assisting the follow-up of its muscle activity during treatments. The use of US has evolved in recent years, becoming an increasingly standardized technique quick of execution, low cost comparable and a feasible and reliable tool.

The knowledge of detailed anatomy of the soleus and gastrocnemius muscles, also named triceps surae, has been reported due to this muscle complex originating on the backside of the femur and attached in conjunction with the soleus muscle by the Achilles tendon at the heel [36,37], as well as strains and activity in both muscles being important for understanding the underlying injury mechanism, a rigorous prognosis, proper treatment and a successful prevention of recurrent injury [8]. The origin of both muscles are anatomically distinct: gastrocnemius arises from above the knee and soleus from below the knee. Bojsen-Moller et al. [38] found that, with the knee flexed, the soleus was the principal generator of force generated in plantar flexion, while, with the knee extended, the gastrocnemius provides the greater force. However, the data of this study showed that the displacement of medial gastrocnemius was lower when the knee was flexed, probably due to the isolation activity of the soleus muscle to perform the force in the movement. The authors hypothesized that, with the knee flexed, if the soleus contraction was lower and the muscle must develop a greater force to perform the plantar flexion, it may be one of the reasons for the injury mechanism of soleus injuries. In this way, the use of M-model US for clinical analysis of the soleus may be the key to determining the activity during normal or injured conditions and guiding the rehabilitation. An US provides advances in clinical practice, as well as in different research lines.

In order to estimate repeated measures of an evaluator with the same tool and differentiate between real change and random measurement errors, the SEM formula was usually employed. Thus, a very small SEM and almost perfect reliability for these measurements were found, with values ranging from 0.020 cm and 0.223 cm/s^2^, the intra-rater thickness at 0 knee degrees variable being the smaller and the intra-rater contraction velocity at 30 knee degrees the highest value. In addition, these results could be argued that the intra-rater thickness reliability at 0 knee degrees presents the lower random measurement error, and the intra-rater contraction velocity at 30 knee degrees reported the highest measurement error in this study. For example, other studies who reported SEM values in order to check the reliability of the ultrasound systems in healthy subjects reported values from 0.02 to 0.06, considering these results as small SEM values [39]. Rosenberg et al. reported 0.720 cm^2^ of SEM value to the cross-sectional area (CSA) in a reliability study of panoramic mode for gastrocnemius muscle [40]. In line with the SEM results, the evaluation with 0 knee flexion degrees also reduced the SRD, while the examination with 30 knee flexion degrees reported a slight increase of the SRD, both SEM and SRD results being reliable parameters for the use of M-mode ultrasound examination of the soleus muscle.

Finally, the study had several limitations. First, the included participants were healthy subjects. It would be of interest to know if similar results would be observed in patients with soleus muscle injuries in athletes. Second, the authors only analyzed the soleus muscle during ankle dorsiflexion. Futures studies could measure the other muscles that participate in that movement to assess their different activations. In addition, future research could compare the M-mode ultrasound imaging and EMG as an inter-method analysis of the soleus muscle and the whole triceps surae complex.

## 5. Conclusions

We found that the M-mode ultrasound examination of the soleus muscle is highly reliable in healthy people, reporting good to excellent an intra- and inter-examiner reliability. Moreover, considering the soleus muscle activity, the results of the present study exhibit lower activity when participants had their knee flexed at 30°. Future studies should investigate if M-mode ultrasound imaging can be employed for the assessment of the soleus muscle in individuals with muscle and soft tissue disturbances in the soleus muscle.

## Figures and Tables

**Table 1:** Sociodemographic data.

Data	Total (*n* = 30)
Age (years)	38.46 ± 2.09
Weight (kg)	67.73 ± 2.00
Height (m)	1.70 ± 0.01
BMI (kg/m^2^)	23.18 ± 0.44

Abbreviations: body mass index, BMI.

**Table 2:** Intra- and inter-rater reliability of the initial thickness, end thickness, contraction time and contraction velocity variables for both positions (knee extended and knee flexed at 30°).

Measures (Units)	ICC (95% CI)	SEM	SRD
0 knee flexion degrees			
Intra-Rater Test Thickness (cm)	0.994 (0.988–0.997)	0.020	0.156
Inter-Rater Test Thickness (cm)	0.911 (0.982–0.996)	0.079	0.619
Intra-Rater MIC (cm)	0.911 (0.822–0.957)	0.088	0.689
Inter-Rater MIC (cm)	0.918 (0.834–0.960)	0.081	0.635
Intra-Rater Contraction Time (s)	0.931 (0.925–0.983)	0.031	0.243
Inter-Rater Contraction Time (s)	0.919 (0.836–0.960)	0.027	0.211
Intra-Rater Contraction Velocity (cm/s^2^)	0.955 (0.906–0.979)	0.097	0.760
Inter-Rater Contraction Velocity (cm/s^2^)	0.810 (0.638–0.905)	0.200	1.568
30 knee flexion degrees			
Intra-Rater Test Thickness (cm)	0.974 (0.947–0.988)	0.035	0.274
Inter-Rater Test Thickness (cm)	0.967 (0.931–0.984)	0.040	0.313
Intra-Rater MIC (cm)	0.867 (0.7402–0.934)	0.100	0.784
Inter-Rater MIC (cm)	0.976 (0.950–0.989)	0.038	0.297
Intra-Rater Contraction Time (s)	0.933 (0.679–0.917)	0.017	0.133
Inter-Rater Contraction Time (s)	0.863 (0.733–0.932)	0.024	0.188
Intra-Rater Contraction Velocity (cm/s^2^)	0.811 (0.641–0.905)	0.223	1.748
Inter-Rater Contraction Velocity (cm/s^2^)	0.899 (0.800–0.951)	0.156	1.223

Abbreviations: ICC, intraclass correlation coefficient; MIC, maximal isometric contraction; SEM, standard error measurement; SD, standard deviation and SRD, smallest real difference.

**Table 3:** Mean and standard deviations of the initial thickness, end thickness, contraction time and contraction velocity variables for both positions (knee extended and knee flexed at 30°).

Measures (Units)	Total Mean (SD)	Dataset 1 Mean (SD)	Dataset 2 Mean (SD)
0 knee flexion degrees			
Intra-Rater Test Thickness (cm)	1.334 (0.26)	1.332 (0.27)	1.336 (0.26)
Inter-Rater Test Thickness (cm)	1.331 (0.26)	1.332 (0.27)	1.330 (0.28)
Intra-Rater MIC (cm)	1.518 (0.29)	1.517 (0.29)	1.519 (0.30)
Inter-Rater MIC (cm)	1.506 (0.28)	1.517 (0.29)	1.494 (0.28)
Intra-Rater Contraction Time (s)	0.182 (0.11)	0.180 (0.10)	0.184 (0.13)
Inter-Rater Contraction Time (s)	0.175 (0.09)	0.180 (0.10)	0.169 (0.08)
Intra-Rater Contraction Velocity (cm/s^2^)	1.123 (0.46)	1.140 (0.48)	1.105 (0.43)
Inter-Rater Contraction Velocity (cm/s^2^)	1.150 (0.46)	1.140 (0.48)	1.161 (0.43)
30 knee flexion degrees			
Intra-Rater Test Thickness (cm)	1.306 (0.22)	1.312 (0.23)	1.300 (0.20)
Inter-Rater Test Thickness (cm)	1.305 (0.22)	1.312 (0.23)	1.297 (0.21)
Intra-Rater MIC (cm)	1.331 (0.29)	1.340 (0.24)	1.314 (0.33)
Inter-Rater MIC (cm)	1.343 (0.25)	1.340 (0.24)	1.339 (0.25)
Intra-Rater Contraction Time (s)	0.121 (0.06)	0.123 (0.66)	0.117 (0.07)
Inter-Rater Contraction Time (s)	0.122 (0.06)	0.123 (0.66)	0.121 (0.06)
Intra-Rater Contraction Velocity (cm/s^2^)	0.781 (0.51)	0.774 (0.49)	0.787 (0.54)
Inter-Rater Contraction Velocity (cm/s^2^)	0.784 (0.49)	0.774 (0.49)	0.793 (0.49)

Abbreviations: MIC, maximal isometric contraction; SEM, standard error measurement and SD, standard deviation.

**Table 4:** Measurement comparison between 0 and 30 knee flexion degrees.

Measures	0 Knee Flexion Degrees	30 Knee Flexion Degrees	*p*-value
Rest thickness	1.33 ± 0.27 (0.77–1.92) *	1.31 ± 0.23 (0.89–1.83) *	0.898 **
MIC thickness	1.51 ± 0.29 (0.21–0.50) *	1.34 ± 0.24 (0.87–1.81) *	0.027 **
Contraction time	0.18 ± 0.10 (0.06–0.60) ^†^	0.11 ± 0.08 (0.01–0.31) ^†^	0.022 ^‡^
Contraction velocity	1.14 ± 0.48 (0.40–2.64) *	0.77 ± 0.76 (0.00–1.82) *	0.006 **

Abbreviations: MIC, maximal isometric contraction * Mean ± standard deviation (SD) (minimum-maximum) was applied. ** The Student *t*-test was performed for independent samples. ^†^ Median ± interquartile range (IR) (minimum–maximum) was used. ^‡^ Mann-Whitney U test was utilized.
